# Goal attainment scaling as an outcome measure in rare disease trials: a conceptual proposal for validation

**DOI:** 10.1186/s12874-019-0866-x

**Published:** 2019-12-04

**Authors:** C. M. W. Gaasterland, M. C. Jansen van der Weide, K. C. B. Roes, J. H. van der Lee

**Affiliations:** 1 Pediatric clinical Research Office, Academic Medical Center, University of Amsterdam, Meibergdreef 9, 1105 AZ Amsterdam, The Netherlands; 2 Pediatric clinical Research Office, Academic Medical Center, University of Amsterdam, Meibergdreef 9, 1105 AZ Amsterdam, The Netherlands; 3 Julius Center, University Medical Center Utrecht, Utrecht, The Netherlands; 4 Pediatric clinical Research Office, Academic Medical Center, University of Amsterdam, Meibergdreef 9, 1105 AZ Amsterdam, The Netherlands

**Keywords:** Goal attainment scaling, Validation, Drug trials, Rare diseases

## Abstract

**Background:**

Goal Attainment Scaling (GAS) is an instrument that is intended to evaluate the effect of an intervention by assessing change in daily life activities on an individual basis. However, GAS has not been validated adequately in an RCT setting. In this paper we propose a conceptual validation plan of GAS in the setting of rare disease drug trials, and describe a hypothetical trial where GAS could be validated.

**Methods:**

We have used the *COnsensus-based Standards for the selection of health Measurement INstruments* (COSMIN) taxonomy to deduce which measurement properties of GAS can be evaluated, and how. As individual GAS scores cannot be interpreted outside the context of a RCT, the validation of GAS needs to be done on trial as well as on individual level.

**Results:**

The procedure of GAS consists of three steps. For the step of goal selection (step 1) and definition of levels of attainment (step 2), face validity may be assessed by clinical experts. For the evaluation of the goal attainment (step 3), the inter and intra rater reliability can be evaluated on an individual level. Construct validity may be evaluated by comparison with change scores on other instruments measuring in the same domain as particular goals, if available, and by testing hypotheses about differences between groups. A difference in mean GAS scores between a group who received an efficacious intervention and a control group is an indication of well-chosen goals, and corroborates construct validity of GAS on trial level. Responsiveness of GAS cannot be evaluated due to the nature of the construct being assessed.

**Conclusion:**

GAS may be useful as an instrument to assess functional change as an outcome measure in heterogeneous chronic rare diseases, but it can only be interpreted and validated when used in RCTs with blinded outcome assessment. This proposed theoretical validation plan can be used as a starting point to validate GAS in specific conditions.

## Background

Goal Attainment Scaling (GAS) [1] is an instrument that is intended for standardized evaluation of the effect of an intervention based on individualized goals. It was originally developed by Kiresuk and Sherman in 1968 to evaluate mental health services. The instrument allows patients to set individual treatment goals, together with their treating professional. The goals need to be defined in such a way that an independent evaluator can assess whether they have been achieved. This means that they must be defined in a measurable, preferably functional domain. The number of goals and the content of these goals may differ per patient, but the attainment of the goals is measured in a standardized way, in the GAS procedure that is similar for every patient.

In the original description, the levels of goal attainment are quantified on a 5-point scale for each goal, ranging from − 2 to + 2. The expected level of goal attainment with the intervention of interest is set at 0; − 1 and − 2 are circumscribed levels somewhat (− 1) or considerably (− 2) below the expected level, and + 1 and + 2 are circumscribed levels somewhat (+ 1) or considerably (+ 2) above this level. The setting of the goals and the definition of the goal attainment levels is decided by the patient or (when the patient is unable to do this) family and their (trained) treating physician. The selected goals and levels of attainment should be defined in a way that complies with the SMART principle: the goals should be Specific, Measureable, Agreed upon, Realistic and Time-related [2]. Currently, GAS is mainly used as an assessment instrument in (pediatric) rehabilitation [2–4], geriatrics research [5, 6], and psychosocial interventions [7].

GAS has been proposed as an instrument to assess the efficacy of potential new drugs in rare disease drug trials [8]. New drugs for rare diseases often have to be tested in small, heterogeneous populations. Existing generic measurement instruments often are not responsive enough to detect the effect of an intervention in a rare disease. Due to the heterogeneity which often occurs in rare disease populations, many measurement instruments have threshold or ceiling effects, or measure constructs that are not relevant for many patients included in the trial. The use of these instruments may lead to an even further reduction of the number of patients who are eligible for a trial. In these specific cases, where the few available patients for inclusion in a trial are quite heterogeneous, and all have different constructs that are relevant to them, GAS may provide a solution. An example of a threshold effect is the use of the 6 Minute Walk Test in a trial in Duchenne’s muscular dystrophy, which can only be applied in patients who are still able to walk. This limits the eligible population to patients below the age of about 10 years, after which most of them are wheelchair bound. The number of rare disease patients is usually insufficient to develop and validate disease-specific measurement instruments. The use of an individualized instrument like GAS may help to alleviate these problems.

An example of a heterogeneous disorder where GAS could be used is mitochondrial disorder. This is a group of rare diseases (prevalence 9.2 in 100,000) with a very wide range of symptoms, such as coordination problems, muscle weakness and hearing loss [9]. Measurement instruments that only focus on one aspect of the disease, such as the 6 min walk test or a hearing test, will only be able to show a potential improvement, or less than expected deterioration, in patients with these particular impairments. A drug for mitochondrial disease that would tackle the underlying disease mechanism could possibly ameliorate all or at least several heterogeneous aspects of the disease. GAS could then be a solution to include all patients in a trial, even when their complaints are all very different. In Table 1, an example of hypothetical goals of mitochondrial disease patients is shown.

**Table 1:** Example of GAS goal topics and scores in (fictitious) patients with mitochondrial disease

Goals	-2 score	0 score (expected outcome level)	+ 2 score
Being able to cook for me and my partner	The patient cooks once a week or less	The patient cooks three times a week	The patient cooks five times a week or more
Being able to watch a film at night without falling asleep	The patient can watch a series episode of 25 min without falling asleep	The patient can watch a series episode of 50 min without falling asleep	The patient can watch a film of 2 h without falling asleep
Being able to dress myself independently	The patient needs help with putting on a shirt and pants	Patient can put on a shirt, but needs help with pants	The patient can dress him/herself without any help

GAS is most useful if improvement can be assessed in functional terms. The key element of GAS is the difference that a patient notices, which can also be assessed more or less objectively by an independent assessor. In some cases this could be measured objectively, even with a validated high quality Patient Reported Outcome Measure (PROM) or function test, but in many cases the patient’s individual goals cannot be captured in an existing validated PROM or other measurement instrument. A patient may find it most important, for example, that he or she is able to see a film without falling asleep. This is a goal that can be defined beforehand, and is easily measurable, but not with a validated measurement instrument. This variety of goals that differ from patient to patient is what makes GAS so responsive and patient-centered, as the chosen goals are inherently relevant to them. Therefore, GAS can only be used if the intervention under evaluation has an expected effect which can be observed in functional status, and it is not suitable to evaluate interventions that have an expected effect only on physiological biomarkers, such as blood pressure or the concentration of particular metabolites in plasma or urine. In the model of Wilson and Cleary five different domains of patient outcome are distinguished, ranging from biological and physiological variables to overall quality of life. The construct measured by GAS is on the level of functional status in this model, thus GAS is only applicable for interventions that have an expected effect on the functional status [10].

GAS cannot be categorized easily as an instrument, since it falls in neither category of self-reported outcomes or assessment instruments. The goals are defined by the patient and the clinician together, and the assessment is preferably done by an independent assessor. Sometimes the underlying construct can be assessed with a PROM, but this is not always the case. This makes GAS different from PROMs. The variable, or construct, that is assessed by GAS is probably best described as the change due to the effect of an intervention on some underlying mechanism of a disease (the ‘intervention effect’) that translates to the patient’s daily life activities in different ways. The operationalization of this construct, which is aimed to be measured by GAS, is the extent to which a goal relevant to the patient and meaningful for the clinician was achieved. This variable is different in every trial, and the content of the goals differs from patient to patient, making GAS different compared to most measurement instruments, which contain one or more unidimensional scales. Consequently, the interpretation of the GAS score is more complex. Although the individual may be able to evaluate whether goals have been attained or not based on the GAS score, the evaluation of an intervention effect based on the GAS score can only be interpreted as a group mean, compared to the mean of another group. This is because a score on its own does not mean anything, as there is no anchor point or ‘reference population’ for comparison; every patient is their own control. Thus, GAS is a relative measure.

GAS may be compared to the Clinical Global Impressions Scale (CGI), which evaluates the overall impression of a patient’s improvement as assessed by clinicians. The CGI can be used in any disorder, and as such does not measure a single construct [11, 12]. The question of validity of the CGI is still topic of discussion [13]. An important aspect of GAS which makes it more objective than the CGI is that goals and levels of goal attainment are defined and recorded in detail beforehand, in such a way that an independent rater can use the patient’s individual scale to assign a score, whereas the CGI can only be assessed by a clinician who has known the patient for a while, with a risk of recollection bias.

Some methodological challenges of GAS are still currently under debate [14, 15]. For example, there is no consensus yet whether GAS scales should be treated as ordinal, how scores from different goals per patient should best be combined, and which statistical tests are valid, although some research on this topic has been carried out [16]. One of the main issues hampering the use of GAS is the lack of validation. GAS has not been adequately validated to be used in drug trials in general [17], or drug trials in rare diseases [8]. However, for GAS to be used as an instrument to evaluate drugs for market authorization, adequate validation has to be put in place [18].

In drug trials aimed at regulatory approval, the preferred primary endpoint is an objectively assessed clinical outcome, such as death. When a particular clinical outcome cannot be used as primary endpoint, for example when the number of events is very small, a surrogate outcome or a biomarker can be used, e.g. blood pressure or viral load [19]. In virtually all trials secondary outcomes are measured as well. According to the EMA HRQL Reflection Paper [20] and the FDA PRO Guidance [21], subjective measurement instruments such as PROMs can also be used as primary or key secondary outcomes in drug studies, provided that these measurement instruments have been validated and the trial is controlled and well-designed. Patient-reported outcomes provide a unique perspective on a treatment benefit, and can therefore also be used in clinical trials, especially in rare diseases [22]. GAS could be particularly relevant in this context. However, validation studies, in which measurement properties such as reliability, validity and responsiveness are evaluated, need to be performed and yield favorable results before a measurement instrument is considered ‘valid’ and could be used in this context. There is no strict guideline on how many of these studies should be performed in order for a measurement or assessment instrument to be considered valid.

In this paper, we propose a conceptual validation plan to evaluate the measurement properties of GAS both on individual level and on a trial level, with the ultimate aim to qualify it as a validated instrument for use in (rare disease) drug trials for regulatory approval, meaning in the setting of a Randomized Controlled Trial (RCT). As a minimum, we propose that the methodological aspects of GAS that can be evaluated have been validated and found adequate.

## Methods

The term ‘Goal Attainment’ or ‘Goal Attainment Scaling’ is sometimes used for instruments or interventions that do not follow the original description by Kiresuk and Sherman. Here we only consider instruments that are in accordance with the original description, translated in the following properties:

- One or more individual goals are established by the patient together with their treating clinician, or by one or more researchers or practitioners, either with or without input of the patient, prior to the intervention. The goals are chosen individually per patient.
- The scale of goal attainment levels has to consist of at least three points (e.g. more than just goal attained – goal not attained). At least 2 points on the scale are described precisely and objectively, so that an independent observer would be able to determine whether the patient performs above or below that point.
- GAS is used as an instrument to evaluate an intervention, and is not used as an intervention itself.

Because GAS is a relative measure, which cannot be interpreted on its own, it can only be evaluated when it is used in a randomized clinical trial where at least the assessors are blinded, and the goals are defined before randomization. Since patients may have an influence on how well goals are attained, they are preferably blinded to treatment also. GAS can only be fully validated at trial level, as the content of goals is different in every patient and the scale cannot be interpreted otherwise. The *COnsensus-based Standards for the selection of health Measurement Instruments* (COSMIN) guidelines, which have been developed to evaluate the methodological quality of studies on measurement properties of HR-PROs and other instruments [23, 24], can be used as a basis for a validation plan. According to these guidelines, quality aspects of a measurement instrument can be divided in three domains, i.e. reliability, validity, and responsiveness.

**Fig. 1:**
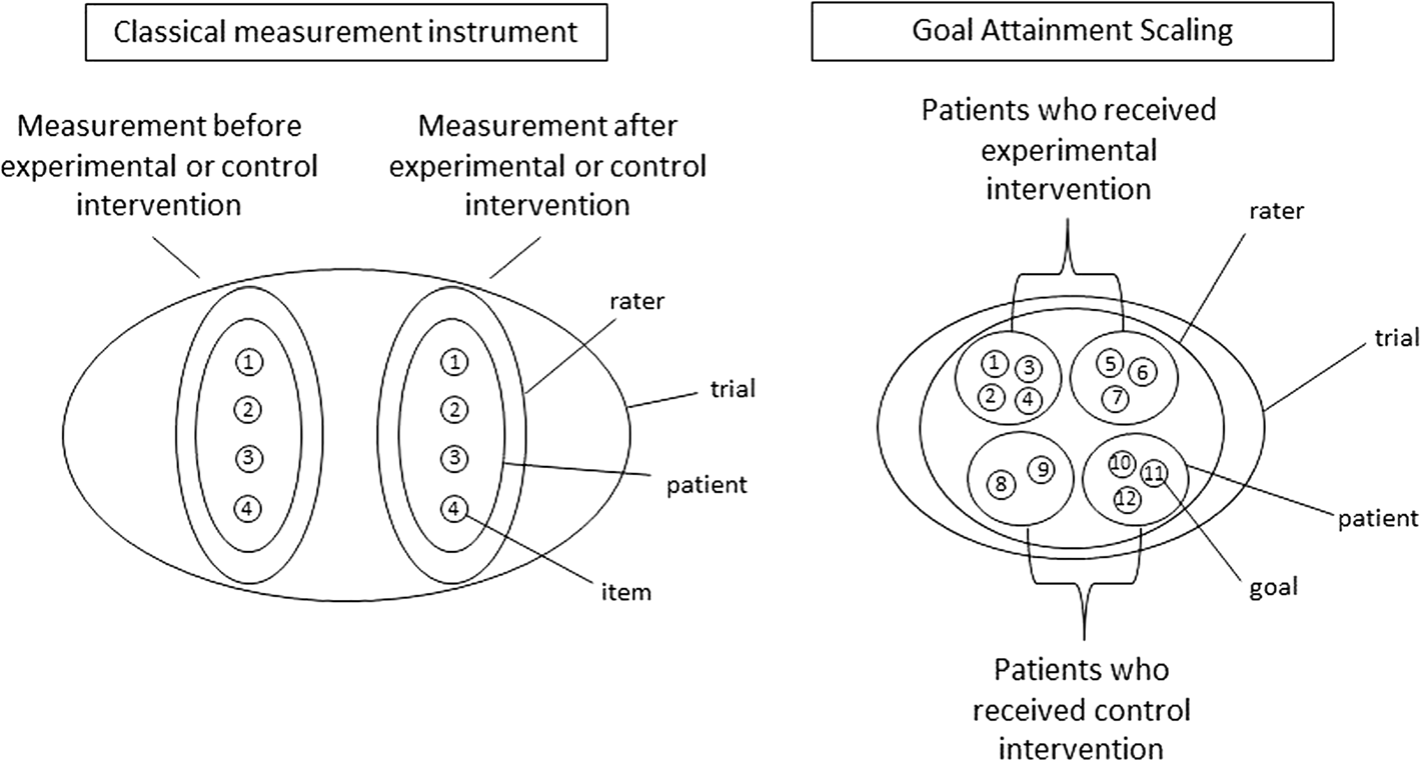
Visualization of the differences between GAS and classical measurement instruments

The COSMIN guidelines were developed for measurement instruments that measure a particular construct or latent variable at a certain points in time, and that can be used to monitor patients over time or as outcome measures in trials. However, GAS measures only the change over time due to the effect of an intervention on some underlying mechanism of a disease that translates to the patient’s daily life activities in different ways. According to the Generalizability Theory in every measurement there are multiple sources of error variance, such as raters or occasions [25]. When using GAS, not only the patients and raters are variable, but also the content of the instrument, as every patient may choose their own goals. This adds an extra dimension, setting GAS apart from classical test theory [26]. Because of the diversity in number of goals, goal content and goal attainment levels among patients the validity and reliability of GAS also need to be assessed on trial level [27]. In Fig. 1, the difference between GAS and classical measurement instruments is visualized. Variation in classical measurement instruments may take place per patient and per rater, but the items remain the same for each measurement. In GAS the goals differ per patient, and there is only one measurement point, which is after the intervention. The most important differences between GAS and other measurement instruments are also listed in Table 2.

**Table 2:** The differences in properties of between GAS compared to other and classical measurement instruments

Classical measurement instruments	Goal Attainment Scaling
Items and scoring options are fixed	Items (goals) and scoring options (goal attainment levels) are different for each patient
Instrument can be used for many patients in many settings	Patients make their own personalized instrument for one occasion together with their clinician
Repeated measurements are possible, for example before and after an intervention, that can be compared	Only one measurement is possible, after an intervention or after a defined period of time
The score is ‘anchored’, e.g. to a general population reference range, or to a cut-off score for a diagnosis, and is interpretable on individual level	The score is not ‘anchored’ and can only be interpreted as a comparison of group means in a randomized setting
One or more unidimensional scales quantifying underlying constructs	Quantifies change due to an intervention (or over time) that is relevant to patients

Because GAS is such a special case, the COSMIN guidelines cannot be applied on GAS without adaptation. We have used COSMIN as a guidance to propose which of the quality aspects could be assessed for GAS in a randomized clinical trial, and what the COSMIN guidelines could mean when used on trial level, but it should be borne in mind that this does not fit perfectly.

There are three domains in the COSMIN taxonomy that can be assessed: reliability, validity and responsiveness. For reliability, we will not only consider the inter- and intra-rater reliability of the individual goal scores, but also the reliability on trial level. Validity can be divided in the content validity of the chosen goals, and the construct validity, which may be assessed per trial, and in comparison with other relevant measurement instruments. Responsiveness of GAS cannot be evaluated in the original sense. The definition of responsiveness as according to COSMIN is the ability of an HR-PRO instrument to detect change over time in the construct to be measured [23]. However, GAS does not measure one construct in one time point, but change on specifically chosen goals over time. Interpretability, a characteristic of a measurement instrument to facilitate its use, also mentioned in the COSMIN taxonomy, is also not directly applicable to GAS. The individual goals and their attainment levels are inherently interpretable for the patients, but the mean GAS scores can only be interpreted by comparison of the experimental and control group scores on a trial level.

## Results

Because the process of GAS consists of three steps, the quality aspects might be assessed for one or more of these three steps: Selecting the goals, defining the levels of attainment, and rating the goal attainment after the intervention.

In the two COSMIN domains validity and reliability, for the three steps of the GAS process, five quality aspects can be evaluated on the individual patient level and two on trial level (see Table 3).

**Table 3:** Proposal for ways to assess aspects of measurement properties of GAS in a blinded placebo-controlled RCT

COSMIN Domain	Quality aspect	COSMIN definition	Definition of quality aspect as applicable to GAS	Measurable on GAS step	How to assess on individual level***	How to assess on trial level
Validity	Content validity	The degree to which the content of an HR-PRO instrument is an adequate reflection of the construct to be measured	The degree to which goals capture patient outcomes that can be expected to be influenced by treatment.	Selecting goals [1]	Evaluation of the goals by independent clinical experts****	
	Content validity	The degree to which the content of an HR-PRO instrument is an adequate reflection of the construct to be measured	Levels are determined consistently, ensuring that they are centered around an appropriate value of zero and proportionally ordered.	Defining the levels [2]	Evaluation of the goal achievement levels by independent clinical experts****	
	Construct validity	The degree to which the scores of an HR-PRO instrument are consistent with hypotheses (for instance with regard to internal relationships, relationships to scores of other instruments, or differences between relevant groups) based on the assumption that the HRPRO instrument validly measures the construct to be measured	The underlying construct (one or more than one for every patient) is the attainment of goals that are chosen to reflect the effect that is expected from an intervention on a functional level. Construct validity is the degree to which the scores of GAS are consistent with hypotheses.	Assessing goal attainment [3]	Hypothesis testing: Comparison of scores on individual goals with change scores on other measurement instruments that measure a construct that is similar to one or more of the chosen goals, such as a function scale or a balance test*	Hypothesis testing: Comparison between two groups. It is expected that the mean GAS scores of two randomized groups receiving effective or non-effective interventions will differ in favor of the group receiving the effective intervention.
Reliability	Intra rater reliability	The extent to which scores for patients who have not changed are the same for repeated measurement by the same persons on different occasions	Goal attainment is assessed consistently when performed by the same rater for the same patient in the same condition repeatedly (possibly hypothetically).	Assessing goal attainment [3]	Repeated assessment of video recording of the assessment of the goal attainment level ** by the same rater	
	Inter rater reliability	The extent to which scores for patients who have not changed are the same for repeated measurement by different persons on the same occasions	Goal attainment is assessed consistently when performed by different raters for the same patient in the same condition.	Assessing goal attainment [3]	Assessment of the video recording of the assessment of the goal attainment level** by one or more independent raters	
	Inter trial reliability		Goal attainment scaling leads to consistent results when implemented in repeated implementations of the same trial.	Assessing goal attainment [3]		Replication between trials or within one trial (with a split-half design); comparison of mean differences between intervention groups

* It is not feasible to compare all chosen goals with other measurement instruments, as goals may vary widely in a heterogeneous population

** In the video recording the goal score should not be made explicit

*** The score of GAS is only a change score, and cannot be interpreted causally at the individual level. However, content validity of GAS can only be assessed on an individual level, since every patient individually chooses his or her own goals. The same goes for reliability and aspects of our proposed construct validity: since all goals differ on an individual level, this has to be assessed at the individual level, in order to ensure the validity and interpretability of GAS on group level

**** *According to the original COSMIN methodology, this should be done very extensively with qualitative interviews and/or focus groups. However, in the setting of rare diseases, this is not feasible*

In the domain of validity, content validity and construct validity can be assessed. The content validity can be evaluated for both selecting the goals and defining the levels of goal attainment. Since the goals have been selected by the patients themselves, the content validity does not need to be evaluated by patients. It can be done by one or more independent clinical experts, who check whether the patient-selected goals are relevant to the intervention (for examples see [28] and [29]). This is very important, as the relationship between the selected goals and the intervention will strongly influence the construct validity of the instrument. In fact, the content validity of GAS in general cannot be assessed, because the content of the goals will be different in every trial, depending on the disease, intervention and also the patients. Because it is of vital importance that goals are selected that are relevant for the intervention, we suggest that a content validity check is performed in every trial where GAS is used, for example by physicians who are able to judge what the effect may be of the intervention on the patient. The chosen goals may be scored on a scale of 1–5. On a trial level, it is important that the variability of selected goals, if present, is comparable in the placebo and the intervention group, which is why blinding and randomization are so important.

Construct validity can be evaluated for the assessment of the levels of goal attainment by comparing (i.e. correlating) the scores for particular goals with change scores on instruments that measure the same construct as those goals, but only for goals that happen to align with other applied measurement instruments. The hypothesis would be that higher GAS scores for a particular goal are associated with larger change scores on an instrument that measures a construct similar to that goal. Since GAS is meant to be applied in situations of inadequate availability of existing measurement instruments, this type of validation will be very limited and haphazard.

Construct validity can also be measured on trial level, theoretically in a (placebo-)controlled trial of an intervention with known efficacy. However, in real life examples, the effect of the intervention will be unknown. This means that when no difference is found between the experimental group and the placebo group, this can either be due to an ineffective intervention, or to lack of responsiveness of the measurement instrument, in the case of GAS: inadequate construct validity. When a difference is found between the experimental group and the placebo group, it can only be concluded that the intervention has an effect and the measurement instrument can detect that effect. This shows that the right goals have been chosen, and is an indication of construct validity on trial level. Replication is important when construct validity is shown in this manner, as it is in all research. However, replication is even harder to achieve in rare diseases, where even in single trials recruitment is a greater challenge than in more frequent diseases. On the other hand, on trial level, replication does not need to be exact replication with the same research question.

In the domain of reliability, specifically the inter- and intra-rater reliability can be measured for the assessment of the levels of goal attainment, provided that this step is video-recorded. One rater (intra-rater reliability) or two different raters (inter-rater reliability) can score the goal attainment levels based on the videotaped interview with the patient or the videotaped function test, and these scores can then be compared. An important condition for this video recording is that the goal attainment score must not be mentioned by the assessor or by the patient (see for example [30]). The domain of reliability should be replicated over several studies to assess the influence of variation between trials. If, as is often the case in rare diseases, the number of expected comparable trials is very small, a split-half design might be used as a substitute for replication in different trials. With several raters the inter- and intra-rater reliability can be evaluated for one half of the patients, and compared to the inter- and intra-rater reliability of the other half of the patients. In this manner, a replication is already done within one trial, and the variation of reliability can be assessed within the same study.

Responsiveness cannot be evaluated for GAS. As mentioned before, responsiveness can be explained as a change of how one scores on a construct over time, but GAS does not measure one construct. Constructs are different from patient to patient, and since GAS already measures change over time, repeating this measurement makes no sense.

**Box 1 Proposal to validate GAS in a trial in Mitochondrial disease**

**Table:** 

GAS might be validated by using it in a double-blind randomized clinical trial with some design additions for validation, for example in a placebo-controlled trial investigating a potential new drug for mitochondrial disease. We will describe a hypothetical trial, that serves as an example to further clarify our validation proposal. In this hypothetical trial protocol, 80 patients are randomly allocated to an experimental or a placebo arm. *Measurements* At baseline the goals are selected and the levels of attainment are defined by each patient together with their treating physician. The expected effect of the intervention and the relevance of the goals for the patient are the main leading factors for this selection. Additional baseline measurements contain the Mitochondrial Disease Adult Scale (NMDAS), a semi-quantitative clinical rating scale that monitors the extensive clinical spectrum of mitochondrial disease [31], and the Berg Balance Scale (BBS) [32], a measurement instrument that measures the construct of balance, which is often impaired for patients with mitochondrial disease. The patients are then randomized to either the experimental or the placebo group, and receive the allocated treatment for the following 12 weeks. Then, after 12 weeks, the assessment of the attainment of the levels takes place. This will be based on a conversation with the patient by an independent assessor, possibly supplemented by an objective measurement (for example, when the goal was defined as ‘I want to walk ten steps’, the number of steps that can be taken consecutively should be measured). The conversation and objective measurement are videotaped. Care is taken that the actually assigned level of goal attainment is not mentioned. Again, the NMDAS and the BBS are performed. *Analysis* For the evaluation of content validity, an external expert on mitochondrial diseases with access to the clinical files of the patients evaluates the content validity of the selected goals and of the attainment levels set at baseline. For the selected goals it is important that they align with the severity of the patient’s disease and impairments. Also, there has to be a relation between the chosen goals and the expected effect of the intervention. The validity of the selected goals and the attainment levels each can be scored on a 5-point scale, 1 indicating low validity and 5 indicating high validity. A mean score of for example 3.5 or more can be considered to denote adequate content validity. Reliability is addressed as follows: The videotaped assessment of the goal attainment is evaluated by two independent assessors to measure the inter-rater reliability [30]. To assess the intra-rater reliability the same assessor will score the videotaped assessment of the goal attainment twice, with at least two weeks’ time in between to preclude recollection. We will consider the reliability adequate when weighted Kappas are 0.60 or more, a score usually considered ‘substantial’ [33]. To assess the inter trial reliability, a split-half design can be used: the weighted Kappas of the first and the last quarter of trial participants are compared with the two middle quarters. To assess construct validity, correlation coefficients are calculated between the GAS scores and the change scores from baseline to follow-up of the NMDAS and the BBS. We expect that there will be a correlation between the goal attainment scores of the patients, and the NMDAS and BBS, since these two validated measurement instruments are often included in studies on mitochondrial disease. The constructs that they measure will probably also be reflected by at least some of the goals chosen by patients with mitochondrial disease. We also expect that GAS may measure some other aspects of the disease that are not captured within these two instruments. Therefore, a moderate correlation is expected (rho between 0.3 and 0.5) between the GAS scores and the change scores of the related other measurement instruments. This will show that GAS measures aspects that are related to the disease, but also something else that is not yet captured by standard measurement instruments. When there is a statistically significant difference in GAS scores between the placebo group and the experimental group, this is considered as an indication of the construct validity of GAS on trial level, and of the efficacy of the new drug.	

## Discussion

In this paper we have explored what validation steps are needed to use GAS as an assessment instrument in rare disease drug trials. We proposed a validation plan for GAS. By following this proposal in an empirical study, we hope that in the near future GAS will prove to be a useful instrument for drug trials in chronic, rare diseases with small and heterogeneous patient populations.

The validation plan of GAS is specifically targeted for GAS as an assessment instrument in randomized controlled studies with blinded outcome assessment. When GAS is used as an instrument for individual evaluation outside a clinical trial, for example to monitor the improvements in a rehabilitation patient, this validation is of less importance. We have targeted our conceptual validation proposal of GAS on a trial level, because on an individual level GAS cannot be interpreted. In this sense, GAS is different from most other measurement instruments, which measure a particular construct or latent variable, whereas GAS quantifies a change score.

Validity of a measurement instrument is not a binary property, although descriptions of instruments in protocols and publications are often limited to phrases like “this instrument has shown adequate validity and reliability” [34]. Results of evaluation of measurement properties in a particular population in a particular setting are often generalized to other settings without much deliberation. The amount of empirical information underlying claims of validity and reliability varies considerably among measurement instruments. Sometimes measurement instruments that have not been validated in exactly the same population as the one included in the trial, are used as instruments for secondary outcome measures in pivotal trials, and this is generally considered adequate by the regulators. For example, in acquired aplastic anemia (AA) and paroxysmal nocturnal hemoglobinuria (PNH), until recently a measurement instrument was generally used to evaluate quality of life which was originally designed for cancer patients and not validated or suitable for AA or PNH patients [35]. In another example, for the approval of a drug for Adrenal Insufficiency (AI) the Psychological General Well-Being Index (PGWB) was used as a measurement instrument to assess well-being as secondary outcome, without any specific validation of the measurement instrument in this population [27]. Once an instrument is considered ‘valid’, it is often widely used, even in situations that are in fact very different from those of the original validation studies. This is more often the case with more general measurement instruments, such as quality of life measures or a Visual Analogue Scale (VAS). Just like for other instruments, some generalization of the evaluation of measurement properties of GAS should be acceptable, at least for similar contexts.

It may be insufficient to evaluate the content validity of GAS in only one trial, since the goals chosen per patient differ in every trial. However, evaluating the content validity for all diseases separately may not be feasible. Some generalization may be accepted after the validity has been shown in several diseases. Other aspects of the validity of GAS may also need to be validated in several diseases, such as construct validity on individual level and on trial level. These parameters can be checked during trials in which GAS is used as an assessment instrument. When GAS has shown to perform reasonably well as an instrument in several studies and over several diseases, the need for more validation studies will become less stringent. In theory, it would be best to evaluate the construct validity of GAS using an intervention of which the efficacy is already known. However, it would not be ethically acceptable to perform an RCT with an intervention that has already shown its efficacy, particularly in rare diseases [36]. This remains one of the key critical points of GAS: it can only really be validated in a trial in which the efficacy of the intervention is known, whereas GAS should actually be used to evaluate whether the intervention makes a difference. If we would like to run such a study with an intervention of known efficacy, this would raise a major ethical point, especially in the context of rare diseases. However, in a setting where the efficacy of an intervention is not known, if no differences between experimental and control arm are found using GAS, this can be due either to the experimental intervention not being efficacious, or to inadequate construct validity of the instrument, e.g. due to choosing the wrong goals. It can also be the case that GAS detects differences between the experimental group and the control group, and another measurement instrument does not, or the other way around. It is likely that GAS will not be used as a primary outcome, especially since not much is known about its validity in trials yet. Then, it is difficult to draw the right conclusion: is this difference due to the (lack of) effect of the intervention, construct validity of GAS or the responsiveness of the measurement instrument used to measure the primary endpoint? This catch− 22 situation may remain a significant challenge for validation research of GAS in the future. The solution to this problem is probably to investigate GAS in several trials, in several different contexts. Then there will indeed be a chance of validating GAS in a trial with an intervention that – after completion of the trial - has shown efficacy on other measurement instruments also.

A distinction can be made between evaluation of the measurement properties of an instrument in general and quality assurance of the use of a measurement instrument in a particular trial. It can be argued that for an instrument like GAS such quality assurance is very important, since so much depends on the proper conduct of the clinician during goal selection and setting the goal achievement levels. Apart from validating GAS in RCTs, consensus is needed on the procedure of GAS for this purpose. For example, not all research groups using GAS presently score the attainment levels the same way. The level of functioning of a patient before the intervention is sometimes taken into account in the GAS-score, by scoring the current level − 1 or − 2. Some researchers have suggested adding an extra − 3 score so that deterioration from the current level of functioning can be scored as well [37, 38]. There are also research groups that suggest using a predefined set of goals that patients can choose from, instead of being able to choose any goal [39]. To minimize the differences between therapists who decide upon the chosen goals together with the patients, standardized GAS courses including certification are recommended [40, 41]. Particularly for the use of GAS in regulatory trials standardization and quality assurance of the assessment process is of paramount importance.

Our proposal for evaluating the validity of GAS is still open for discussion. It is possible that researchers choose different approaches in validating GAS as an assessment instrument in rare disease trials. However, we think that this proposal may be a good starting point. Some of the proposed validity assessments have already been executed. For example, in a study that evaluated the use of Fluvoxamine in panic disorder and agoraphobia patients GAS was used as a primary outcome measure and ‘face validity’ was assessed by independent raters who scored the relevance of the chosen goals [42]. The goals were considered to be ‘suitably chosen’, which corroborates the ‘face validity’, in our opinion the content validity, of GAS in this setting.

## Conclusion

To conclude, based on what we have proposed in this paper, we think it is possible to validate GAS, which would be a prerequisite to further stimulate its use. GAS assesses change that is inherently relevant to patients, and its individualized approach makes the instrument appealing for small, heterogeneous groups. Although there are still some steps that need to be taken to determine the validity of GAS, application of this proposal for validating GAS will help to enable the performance of studies that are currently not feasible due to a lack of relevant and responsive measurement instruments. The steps that need to be taken should not just be theoretical, but mainly empirical; therefore, we propose that GAS is taken up as an additional measurement instrument in trials, specifically in a context where GAS may be most useful, such as the field of rare diseases.

## Data Availability

Not applicable.

## Abbreviations

AA: Aplastic Anemia
AI: Adrenal Insufficiency
BBS: Berg Balance Scale
CGI: Clinical Global Impressions Scale
COSMIN: COnsensus-based Standards for the selection of health Measurement INstruments
GAS: Goal Attainment Scaling
NMDAS: Mitochondrial Disease Adult Scale
PGWB: Psychological General Well-Being Index
PNH: Paroxysmal Nocturnal Hemoglobinuria
PROM: Patient Reported Outcome Measure
RCT: Randomized Controlled Trial
VAS: Visual Analogue Scale
